# Does Gender Influence the Indication of Treatment and Long-Term Prognosis in Severe Aortic Stenosis?

**DOI:** 10.3390/jcdd10020038

**Published:** 2023-01-22

**Authors:** Celia Gil Llopis, Amparo Valls Serral, Ildefonso Roldán Torres, Maria Belén Contreras Tornero, Ana Cuevas Vilaplana, Adrian Sorribes Alonso, Pablo Escribano Escribano, Pau Gimeno Tio, Esther Galiana Talavera, Juan Geraldo Martínez, Paula Gramage Sanchis, Alberto Hidalgo Mateos, Vicente Mora Llabata

**Affiliations:** Department of Cardiology, Hospital Universitario Dr Peset, 46017 Valencia, Spain

**Keywords:** aortic valve stenosis, sex differences, aortic valve replacement, mortality, treatment outcome

## Abstract

Introduction: It is a matter of controversy whether the therapeutic strategy for severe aortic stenosis (AS) differs according to gender. Methods: Retrospective study of patients diagnosed with severe AS (transvalvular mean gradient ≥ 40 mmHg and/or aortic valvular area < 1 cm^2^) between 2009 and 2019. Our aim was to assess the association of sex on AVR or medical management and outcomes in patients with severe AS. Results: 452 patients were included. Women (51.1%) were older than men (80 ± 8.4 vs. 75.8 ± 9.9 years; *p* < 0.001). Aortic valve replacement (AVR) was performed less frequently in women (43.4% vs. 53.2%; *p* = 0.03), but multivariate analyses showed that sex was not an independent predictor factor for AVR. Age, Charlson index and symptoms were predictive factors (OR 0.81 [0.82–0.89], OR 0.81 [0.71–0.93], OR 22.02 [6.77–71.64]). Survival analysis revealed no significant association of sex within all-cause and cardiovascular mortalities (log-rank *p* = 0.63 and *p* = 0.07). Cox proportional hazards analyses showed AVR (HR: 0.1 [0.06–0.15]), Charlson index (HR: 1.13 [1.06–1.21]) and reduced LVEF (HR: 1.9 [1.32–2.73]) to be independent cardiovascular mortality predictors. Conclusions: Gender is not associated with AVR or long-term prognosis. Cardiovascular mortality was associated with older age, more comorbidity and worse LVEF.

## 1. Introduction

Aortic stenosis (AS) is the most common valvular heart disease in developed countries, which particularly affects the elderly [1]. Sex differences have been detected in the pathophysiology of AS; women present a less calcified and more fibrotic aortic valve during echocardiography, a smaller annulus and a lower coronary height than men, in addition to displaying differences in the left ventricular (LV) remodeling pattern and the adaptative response to pressure overload [2,3,4,5,6]. Previous observational studies using transthoracic echocardiography have shown that women present a more concentric LV hypertrophy, with smaller cavities, greater relative wall thickness and commonly supernormal LV ejection fraction (LVEF) [7,8]. In contrast, recent cardiac magnetic resonance studies have demonstrated more concentric LV remodeling in men [9]. This has implications for clinical presentation, choice of treatment and outcome.

Aortic valve replacement (AVR) is the class I recommendation for symptomatic patients and asymptomatic patients with LVEF < 50% [10]. However, gender differences have been observed with respect to referral and outcomes. Women are usually older and have more severe symptoms than men at the time of diagnosis [11,12]. The literature provides varying conclusions regarding the correlation of female sex on early outcomes of surgical AVR [13,14,15,16], though most studies demonstrate a lower survival rate in women [12,17,18,19].

The aim of this study was to assess the correlation of sex on clinical presentation, treatment and long-term outcomes in AS patients in our health department.

## 2. Methods

### 2.1. Patient Population

Our population was a retrospective cohort (*n* = 452) of patients ≥ 18 years old diagnosed with severe AS (transvalvular mean gradient ≥ 40 mmHg and/or AVA < 1 cm^2^) in our health department between April 2009 and September 2019, with AVR indication according to current clinical guidelines. Exclusion criteria were: (a) subvalvular or supravalvular stenosis; (b) any prior valve operation (surgical or percutaneous); (c) congenital heart disease (except bicuspid aortic valve); and (d) being on the waiting list for AVR.

Demographic data, clinical characteristics and the events in the follow-up were extracted from electronic medical records. The electronic data belonged to a public development clinical care information system. The following data were collected: age, sex, hypertension (HTN) (patients on antihypertensive medications or known to have hypertension, but not receiving treatment [blood pressure ≥ 140/90 mmHg]), diabetes mellitus (patients on oral hypoglycemic or insulin medications and/or HbA1c ≥ 6.5%), hyperlipidemia (patients on lipid-lowering medication), chronic kidney disease (CKD) (estimated glomerular filtration rate (eGFR) ≤ 60 ml/h/1.73 m^2^), atrial fibrillation (AF), anemia (Hb < 12 g/dL for women and Hb < 13 g/dL for men), pacemaker carriers, coronary artery disease (CAD) (or a history of myocardial infarction, coronary artery bypass graft or percutaneous coronary intervention), chronic obstructive pulmonary disease (COPD), current smoking habit and pharmacotherapy. Patients were considered symptomatic if they presented with angina, dyspnea or syncope, which was considered by the evaluating physician to be secondary to AS.

The study was conducted in accordance with institutional policies, national legal requirement, and the revised principles of the Declaration of Helsinki.

### 2.2. Echocardiography

Echocardiographic data were retrospectively obtained by reviewing the electronic medical records of patients with a clinical diagnosis of AS. All patients had severe AS, defined as a mean transvalvular gradient ≥ 40 mmHg and/or aortic valvular area (AVA) < 1 cm^2^, according to standardized guidelines [20,21,22].

Among the variables considered, paradoxical low-flow low-gradient (LF-LG) severe AS was defined as AVA ≤ 1.0 cm^2^, mean transvalvular gradient < 40 mmHg, and LVEF ≥ 50%. Reduced LVEF was defined as ≤40%, LV hypertrophy was confirmed when the septum > 12 mm, concentric remodeling was defined as a relative parietal thickness ≥ 0.45 and moderate pulmonary hypertension (PH) was categorized as sPAP ≥ 40 mmHg.

### 2.3. Follow-Up and Endpoints

The inception date for time-to-event models was that of the first echocardiogram to reveal severe AS, and follow-up continued until death or end of follow-up date (February 2021). Given the retrospective nature of the study, informed consent was waived.

Treatment of AS (AVR or conservative treatment) was initially indicated by a clinical cardiologist according to the European Society of Cardiology guidelines [22,23,24]. The patients who were eventually referred for AVR were evaluated by the heart valve team who decided final treatment strategy: AVR surgery with mechanical or biological prosthesis, TAVR or conservative treatment. Surgical risk was estimated with the EuroScore II and the presence of comorbidity was calculated using the Charlson Index.

The primary end point was the association of sex on cardiovascular mortality (including cardiac and stroke mortality) during follow-up. The secondary end point was to analyze sex differences in the choice of treatment strategy (AVR vs. conservative treatment).

Treatment strategy and date and nature of death were confirmed by reviewing electronic medical records. When the current status of patients could not be determined, national death databases were consulted.

### 2.4. Statistical Analysis

Continuous variables are expressed as mean and standard deviation (SD) in the case of normal distribution, median and interquartile range 25–75 if distribution is not symmetric, and percentages for categorical variables and proportions. Odds ratios (OR) and hazard ratios (HR) are presented with 95% confidence intervals (95 CI). Continuous data were tested for normality of distribution and homogeneity of variance with Kolgomorov–Smirnov and Levenne tests, respectively. To compare clinical, echocardiographic, and laboratory characteristics between women and men’s continuous variables were compared using a Student’s t test and the categorical variables were compared by χ^2^ test.

A multivariate binary logistic regression analysis, with backward stepwise selection method, was performed to detect those that were independently associated with the AVR indication. The following variables were introduced: age, sex, diagnosis before 2011, Charlson index, EuroScore II index, COPD, DM, CKD, eGFR, AF and symptoms. We included the date of diagnosis prior to 2011 as a variable because TAVR was implanted in our center that year. For model building, we selected variables with significant statistical association or those that showed a trend (*p* < 0.10) with respect to AVR indication. We use the likelihood ratio to determine the significance of each model.

The survival-free rates of cardiovascular and all-cause mortalities at follow-up were estimated using the Kaplan–Meier survival curves and comparison between groups (gender and type of the treatment) with the log-rank test (Mantel–Cox). To identify the independent predictors of cardiovascular mortality, potential predictors’ variables with a *p* < 0.10 in the univariant analysis were included in the analysis (sex, age, Charlson index, AVR, reduced LVEF, AF, COPD, CKD and eGFR), which were incorporated with a stepwise selection procedure into a multivariable Cox regression proportional hazards model. Values of *p* < 0.05 were considered statistically significant. Statistical analyses were performed using SPSS version 18.0 software (IBM, Armonk, NY, USA) and MedCalc^®^ Statistical Software version 20.014 (MedCalc Software Ltd., Ostend, Belgium, 2021).

## 3. Results

### 3.1. Baseline Characteristics

In our cohort, women (51%) were older than men (80 ± 8.4 vs. 75.8 ± 9.9 years; *p* < 0.001). The prevalence of CAD and COPD was lower in women than in men (18.6% vs. 33% and 6.5% vs. 23.9%; *p* < 0.001 for both). There were no significant differences between sexes in cardiovascular risk factors, such as HTN, DM, hyperlipidemia, CKD, anemia or AF. There was no difference in the rate of symptomatic patients (93.5% vs. 90.5%; *p* = 0.47), but women presented dyspnea more frequently (83.5% vs. 75.5%; *p* = 0.04) and angina less frequently (22.1% vs. 31.7%; *p* = 0.02) than men. The distribution of sex was similar among pharmacologically treated patients at the time of diagnosis, but women received more ACE/ARBs and fewer ASA (Table 1).

The AVA was lower in women (0.65 ± 0.19 vs. 0.73 ± 0.16; *p* < 0.001), while there were no significant sex differences in the mean gradient (Table 2). Women’s hearts were also smaller, with less myocardial mass, smaller aortic root and smaller LV end-diastolic diameter. There was no significant difference in the frequency of LV hypertrophy between both sexes, but concentric remodeling was more frequent among women. LVEF was higher in women, among whom the percentage presenting LVEF > 50% was higher than men (Table 2). The incidence of PH was higher in women (55.8% vs. 44.7%; *p* = 0.002). The prevalence of paradoxical LF-LG severe AS did not differ significantly between the genders (12.5% vs. 14.9%; *p* = 0.19).

### 3.2. Surgical Risk and Aortic Valve Replacement

Of the total, 251 patients were referred for AVR (surgical or TAVR). The indication of AVR was higher among men (50.2% vs. 60.1%; *p* = 0.02). Subsequently, 17 patients (12 women vs. 5 men; *p* = 0.1) refused to undergo the procedure and 20 patients (9 women vs. 11 men; *p* = 0.57) were rejected from the procedure due to surgical risk and comorbidities. Thus, AVR was eventually performed in 108 women and 125 men (46.7% vs. 56.5%; *p* = 0.03). Surgical AVR was the most prevalent treatment option in both genders but was more frequent among men (80% vs. 74.1%; *p* = 0.02). The rate of biological prosthesis implant was similar in both genders, while that of mechanical prosthesis implant was lower in women. No significant differences were observed in the choice of TAVR in function of sex. Transfemoral access was the most frequent in both genders, while transaxillary access was more frequent in men (Table 3).

Combined surgery (with coronary revascularization or another valve intervention) was performed in 13.7% of patients and was more frequent in men (32% vs. 19.4%; *p* = 0.005), at the expense of coronary artery bypass surgery (24.8% vs. 12.9%; *p* = 0.02). The therapeutic strategy for paradoxical LF-LG severe AS did not differ significantly between men and women. Among the periprocedural complications reported during follow-up, only stroke was significantly higher in women (8.3% vs. 2.4%; *p* = 0.04). Perioperative deaths did not differ significantly between the genders (Table 3).

**Figure 1:**
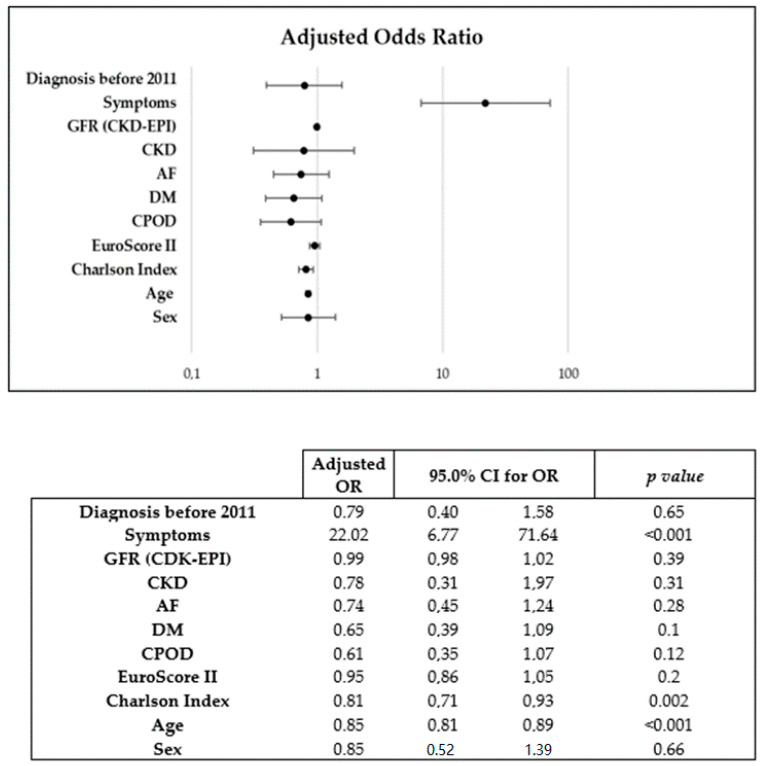
Binary logistic regression model used for multivariate analysis of predictors of AVR.

Sex was a predictive factor for an AVR in the univariate (OR: 0.67 [0.47–0.98]; *p* = 0.04) but not in the multivariate analysis (OR: 0.85 [0.52–1.31]; *p* = 0.62). Older age (OR: 0.85 [0.81–0.89]; *p* < 0.001) and higher Charlson Index score (OR 0.81 [0.71–0.93]; *p* = 0.002) were associated with a lower occurrence of AVR, and the presence of symptoms (OR 22.02 [6.77–71.64]; *p* < 0.001) was the only variable that was directly related to AVR (Figure 1).

### 3.3. Impact of Sex on Clinical Outcomes

**Figure 2:**
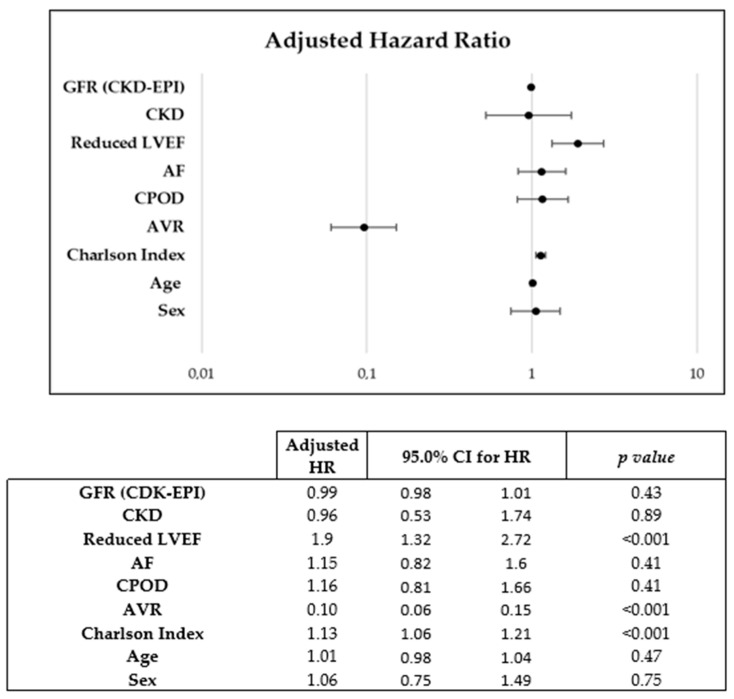
Cox regression used for multivariate analysis of predictors of cardiovascular mortality.

**Figure 3:**
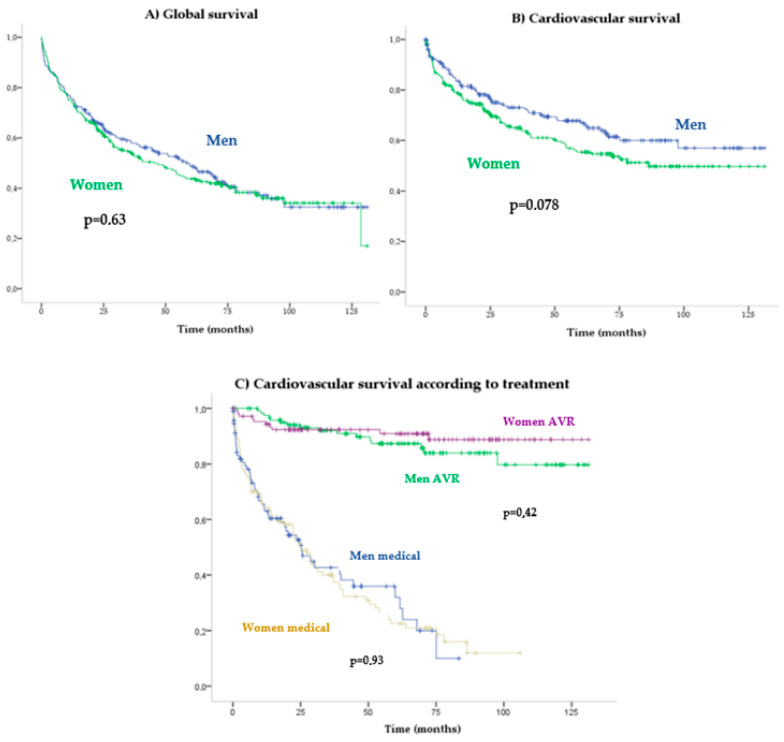
Kaplan–Meier survival curves according to gender.

During a mean follow-up of 2.4 (4.78) years, 254 patients died, of which 54.2% were women. A Kaplan–Meier mortality analysis did not reveal a significant association between sexes in all-cause (Log-Rank *p* = 0.63) (Figure 3A) or cardiovascular (Log-Rank *p* = 0.07) (Figure 3B) mortality. Sex was not found to be associated with cardiovascular mortality in the Cox multivariate analysis (HR: 1.06 [0.75–1.49]; *p* = 0.748) in which the following mortality predictors were detected: AVR (HR 0.1 [0.06–0.15]), Charlson index (HR 1.13 [1.06–1.21]) and reduced LVEF (HR 1.9 [1.32–2.73]) (Figure 2).

**Figure 4:**
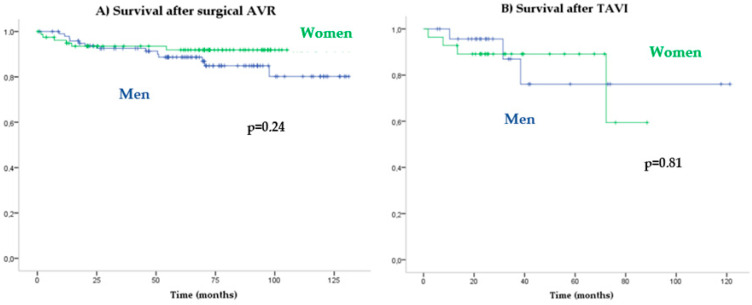
Kaplan–Meier cardiovascular survival according to type of AVR.

Patients with AVR had higher survival rates than with medical treatment (HR: 0.08 [0.05–0.13]; *p* < 0.001). No significant differences in cardiovascular mortality were observed between the genders in the function of the treatment received: conservative treatment (log-rank *p* = 0.93) versus AVR (log-rank *p* = 0.42) (Figure 3C). Long-term prognosis after surgical AVR (log-rank *p* = 0.24) (Figure 4A) versus TAVR (log-rank *p* = 0.81) also did not differ significantly between women and men (Figure 4B).

## 4. Discussion

In our cohort of patients diagnosed with severe AS and treated according to routine clinical practice, we observed these main results: 1. AS was diagnosed later in women, in whom there was a higher prevalence of dyspnea; 2. AVA and heart dimensions were lower in women, while there were no differences between the two sexes in mean aortic transvalvular gradient; 3. AVR indication was more frequent in men than in women, but gender was not an independent predictive factor in the performance of AVR; and 4. Patients treated with AVR had similar survival rates, independently of sex. These data add to previous evidence of similar rates of interventional management and outcomes in both genders [13,25].

Sex differences exist in cardiac form and function, so prevalence, age at diagnosis and severity of symptoms of cardiomyopathies could be sex-specific [26]. AS produces an increase in cardiac afterload that favors ventricular remodeling. Similarly to that described in previous reports [2,4,7,8], the left ventricular cavities of our female patients were smaller and presented greater concentric remodeling and higher LVEF than those of our male patients. On the one hand, this type of remodeling will prolong the phase of clinical stability due to left ventricular compensation of pressure overload. This would partly explain why women are diagnosed later than men; indeed, in our study they were an average of 5 years older. On the other hand, concentric remodeling alters diastolic function and causes intraventricular pressure overload, which that may have been related to the higher rates of dyspnea and PH observed among our female patients. In this sense, other reports have affirmed that women suffer more severe symptoms at the time of diagnosis [11,12,13], though we did not observe this in our female population.

Some studies have supported the critical need for sex-specific criteria to diagnose heart disease, such as dilated and hypertrophic cardiomyopathies, arguing that the use of non-specific diagnostic criteria have a significant negative impact on women [27,28,29]. In the case of AS, a higher prevalence of paradoxical LF-LG AS has been described in females [30,31], though we did not find sex differences in our analysis. It is possible that LF-LG AS is underdiagnosed in women during daily clinical practice. In our study, hemodynamic parameters of the severity of AS were comparable in both sexes, while AVA was lower in women. This could have been due to smaller heart size rather than more severe stenosis. Another possible explanation is that women are in a progressed phase of the disease at the time of diagnosis because of the underdiagnosis of LF-LG AS. In this sense, there may be a need to reformulate the diagnostic criteria for AS in women so as to give more importance to AVA and the dimensionless index.

Delays in the diagnosis of AS and the underdiagnosis of symptoms in women have a negative impact on their prognosis. Moreover, there is controversy about whether the therapeutic strategy for severe AS differ according to gender. Due to their older age, greater comorbidity and higher AS severity, fewer women tend to be referred for AVR, and this has been related to higher long-term morbidity and mortality in several registers [2,3,11,12,30,31,32]. In line with the above, a recent study by the Quebec Heart and Lung Institute highlighted that women were referred for AVR less frequently than men and attributed a higher mortality rate to a lower rate of AVR in paradoxical LF-LG AS [16].

In our patients, 50.2% of women and 61.1% of men were referred for intervention, despite there being no differences between the two genders in terms of the presence of symptoms, Charlson comorbidity Index or EuroScore II. Sex was not an independent predictor factor for AVR, but older age did determine a less frequent indication of AVR among women. In this sense, the progressive increase in TAVR among older patients may have mitigating differences in the frequency of AVR indication among females.

Previous studies have reported worse surgical results in women due to higher mortality in the first 30 days post-AVR. In our cohort, surgical AVR was predominant in men but we did not observe differences in survival rates after AVR (surgical or transcatheter) according to gender (Figure 3A,B). Perioperative complications (including perioperative death, bleeding, infection, cardiac tamponade, cardiac rupture, suture dehiscence, need of pacemaker and others) were not more frequent among our females, though they did display a higher rate of stroke, as reflected by other studies [17,33].

Global prognosis did not differ between women and men, thus supporting previous research that did not detect poorer outcomes in women [13,25]. The discrepancy between our data and that of other registers might be explained by two factors: first, better criteria for the selection of candidates for AVR, regardless of the fact that women debut older and with more severe symptoms; second, the underdiagnosis of paradoxical LF-LG AS, which is less frequently related to AVR [34,35,36,37].

## 5. Limitations

This was a single-center observational study, which limits any causal interpretation. The retrospective nature of our study means that the data collection was less exhaustive than in a prospective study. During the recruitment period there may have been changes in treatment protocols that could have influenced the results. The total number of valve replacement interventions has increased since the introduction of TAVR; however, our multivariate analysis indicated that the year of AS diagnosis after 2011 was not related to AVR (Figure 1).

The presence of frailty or other life-threatening comorbidities not included in the Charlson comorbidity index and the EuroScore II might have influenced decisions on whether or not to perform AVR in our population.

## 6. Conclusions

In our work, women were diagnosed with AS at an older age than men. AVR was indicated less frequently in women, though gender was not an independent predictive factor for AVR. Regardless of sex, cardiovascular mortality was higher among older patients with more comorbidities and low LVEF. Gender was not an independent predictive factor of AVR outcome.

Key Points:

What is known about the subject?

Women are usually referred for AVR less frequently than men, probably due to their older age and more severe symptoms at diagnosis.

Surgical AVR outcomes are worse among women in populations with similar surgical risk.

What do our data add to the knowledge?

Sex was not a predictive factor for AVR during long-term follow up.

Long-term outcomes and perioperative death after surgical AVR did not differ between sexes.

## Figures and Tables

**Table 1:** Baseline clinical data.

	Whole Cohort *n* = 452	Men *n* = 221 (48.9%)	Women *n* = 231 (51.1%)	*p*
Age (years)	77.9 ± 9.4	75.8 ± 9.9	80 ± 8.4	<0.001
Charlson Index	6.2 ± 2.4	6.2 ± 2.7	6.3 ± 2.2	0.65
Charlson Index > 5	339 (75%)	157 (71%)	182 (78.8%)	0.06
EuroScore II Index	3.8 ± 3.2	3.6 ± 3.1	3.9 ± 3.4	0.26
Chronic Kidney disease	180 (39.8%)	79 (35.7%)	101 (43.7%)	0.08
Hypertension	333 (73.7%)	156 (70.6%)	177 (33.3%)	0.14
Diabetes Mellitus	178 (39.4%)	97 (43.9%)	81 (35%)	0.05
Dyslipidemia	235 (52%)	121 (54.7%)	114 (49.3%)	0.25
Anemia	229 (50.7%)	112 (50.7%)	117 (50.6%)	0.99
Chronic pulmonary disease	116 (25.7%)	73 (33%)	43 (18.6%)	<0.001
Atrial fibrillation/flutter	140 (31%)	66 (29.8%)	74 (32%)	0.68
Coronary artery disease	68 (15%)	53 (23.9%)	15 (6.5%)	<0.001
Symptoms	416 (92%)	200 (90.5%)	216 (93.5%)	0.47
Dyspnea	360 (79.6%)	167 (75.5%)	193 (83.5%)	0.04
Angina	121 (26.8%)	70 (31.7%)	51 (22.1%)	0.02
Syncope	55 (12.2%)	28 (12.6%)	27 (11.7%)	0.72
Pharmacotherapy				
Diuretics	279 (61.7%)	125 (56.6%)	154 (66.6%)	0.05
Beta blockers	168 (37.1%)	89 (40.2%)	79 (34.2%)	0.12
ACE/ARBs	213 (47.1%)	92 (41.6%)	121 (52.4%)	0.04
Calcium channel blockers	92 (20.3%)	45 (20.4%)	47 (20.3%)	0.89
Oral anticoagulants	142 (31.4%)	63 (28.5%)	79 (34.1%)	0.25
Acetylsalicylic acid	138 (30.5%)	82 (37.1%)	56 (24.2%)	0.002

**Table 2:** Echocardiography data.

	Whole Cohort *n* = 452	Men *n* = 221 (48.9%)	Women *n* = 231 (51.1%)	*p*
LV ejection fraction (%)	57.3 ± 11.4	55.6 ± 12.7	58.9 ± 9.7	0.002
LV ejection fraction ≤ 40%	99 (21.9%)	63 (28.5%)	36 (15.6%)	0.001
Transaortic mean pressure gradient (mmHg)	48.4 ± 12.2	48.7 ± 12.8	48.1 ± 11.6	0.57
Transaortic maximum pressure gradient (mmHg)	80.6 ± 19.8	78.7 ± 18.6	82.4 ± 20.7	0.05
Peak aortic jet velocity (m/s)	4.4 ± 0.6	4.3 ± 0.4	4.4 ± 0.7	0.55
Aortic area valve (cm^2^)	0.69 ± 0.17	0.73 ± 0.16	0.65 ± 0.19	<0.001
Aortic root (mm)	30 ± 4.6	31.2 ± 4.3	29 ± 4.6	<0.001
sPAP (mmHg)	48.5 ± 14.7	47.9 ± 13.5	49 ± 15.6	0.5
Myocardial mass (g)	250.2 ± 74.5	270.6 ± 81.7	231.1 ± 61.4	<0.001
LV end-diastolic (mm)	47.2 ± 6.9	49.7 ± 7	44.8 ± 5.9	<0.001
Septum (mm)	14.3 ± 2.4	14.3 ± 2.4	14.3 ± 2.3	0.63
Posterior wall (mm)	12.4 ± 2.1	12.3 ± 2	12.4 ± 2.2	0.96
At least moderate pulmonary hypertension	228 (50.4%)	99 (44.7%)	129 (55.8%)	0.02
LV hypertrophy	349 (77.2%)	170 (76.9%)	179 (77.5%)	0.88
Concentric remodeling	351 (77.6%)	159 (71.9%)	192 (83.11%)	0.004
At least moderate aortic regurgitation	36 (8%)	20 (9%)	16 (6.9%)	0.39
Paradoxical LF-LG severe AS	62 (13.7%)	33 (14.9%)	29 (12.5%)	0.19

**Table 3:** Treatment according to sex.

	Men *n* = 221 (48.9%)	Women *n* = 231 (51.1%)	*p*
**Indicated treatment**			
Conservative treatment	86 (38.9%)	115 (49.8%)	0.02
AVR	135 (61.1%)	116 (50.2%)	
**Performed treatment**			
Conservative treatment	96 (43.4%)	123 (53.2%)	0.03
AVR	125 (56.6 %)	108 (46.7%)	
Surgical AVR	100 (80%)	80 (74.1%)	0.02
Mechanical prosthesis	75 (60%)	69 (63.9%)	0.35
Biological prosthesis	25 (20%)	11 (10.2%)	0.01
TAVR	25 (20%)	28 (25.9%)	0.78
Transfemoral access	15	26	0.09
Transapical access	4	1	0.17
Transaxillary access	6	1	0.04
Associated coronary revascularization	31 (24.8%)	14 (12.96%)	0.02
Combined surgery (+ coronary/another valve)	40 (32%)	21 (19.44%)	0.005
Urgent transfer between centers	25 (20%)	12 (11.11%)	0.07
Complication during surgery	14 (11.2%)	12 (11.11%)	0.98
Reoperation	6 (4.8%)	6 (5.55%)	0.68
Post-procedure stroke	3 (2.4%)	9 (8.33%)	0.04
Death on waiting list	8 (6.4%)	6 (5.55%)	0.53
Death during procedure	8 (6.4%)	11 (10.18%)	0.28
Perioperative death (30 days)	7 (5.6%)	11 (10.18%)	0.18
Time since diagnosis to AVR (days)	342.45 ± 367.6	330.9 ± 330.8	0.8
**Paradoxical LF-LG severe AS**			
Conservative treatment	17 (51.5%)	14 (53.3%)	
AVR	16 (48.5%)	14 (46.6%)	
Mechanical prosthesis	2 (12.5%)	1 (7.14%)	
Biological prosthesis	12 (75%)	10 (71.4%)	
TAVR	2 (12.5%)	3 (21.4%)	

## Data Availability

Not applicable.

## Abbreviation

AS: aortic stenosis; AVR: aortic valve replacement; AVA: aortic valve area; LVEF: left ventricular ejection function. ACE: angiotensin I-converting enzyme; ARBs: inhibitors or angiotensin II receptor blockers; LV: left ventricular; sPAP: systolic pulmonary arterial pressure; LF-LG: low flow–low gradient; TAVR: transcatheter aortic valve implant; LF-LG: low flow–low gradient; GFR: glomerular filtrate ratio (ml/min/1.73m^2^); CKD: chronic kidney disease; AF: atrial fibrillation; DM: diabetes mellitus; CPOD: chronic pulmonary obstructive disease; LVFE: left ventricular ejection fraction; AF: atrial fibrillation.
